# Range wide molecular data and niche modeling revealed the Pleistocene history of a global invader (*Halyomorpha halys*)

**DOI:** 10.1038/srep23192

**Published:** 2016-03-21

**Authors:** Geng-Ping Zhu, Zhen Ye, Juan Du, Dan-Li Zhang, Ya-hui Zhen, Chen-guang Zheng, Li Zhao, Min Li, Wen-Jun Bu

**Affiliations:** 1 Tianjin Key Laboratory of Animal and Plant Resistance, College of Life Sciences, Tianjin Normal University, 393 Binshui Road, Tianjin 300387, China; 2 College of Life Sciences, Nankai University, 94 Weijin Road, Tianjin, 300071, China

## Abstract

Invasive species’ Pleistocene history contains much information on its present population structure, dispersability and adaptability. In this study, the Pleistocene history of a global invasive pest (Brown Marmorated Stink Bug BMSB, *Halyomorpha halys*) was unveiled using the coupled approach of phylogeography and ecological niche modelling. Rangewide molecular data suggests that the Taiwan and other native populations had diverged in mid-Pleistocene. In mainland China, the native BMSB did not experience population contraction and divergence during last glacial, but persisted in interconnected populations. Combined Bayesian Skyline Plot (BSP) and niche modelling revealed a rapid expansion occurred during the transition of Last Inter Glacial (LIG) to Last Glacial Maximum (LGM). High genetic diversity and multi-reticular haplotypes network exist in the original sources populations of BMSB invasion in northern China. They were speculated to be colonized from the central China, with many derived haplotypes evolved to adapt the novel environment. The ENM future prediction suggest that BMSB may expand northward to higher latitudes in the US and Europe, because of its high invasive ability, together with the available suitable climate space there.

Invasive species’ Pleistocene history contains much information on its present population structure, dispersability and adaptability^1^. Pleistocene climatic fluctuations are thought to have profound effects on the geographic distribution and genetic diversity of extant species^2^. Recent phylogeographic studies have shed great light on where species persisted during glacial phases and on the routes of post glacial recolonization^3^. However, our knowledge is uneven due to the number of studies conducted in different regions across the globe and also to the type of species (e.g., vertebrate and plant versus invertebrate) considered. Pleistocene climatic fluctuations in East Asia were less extensive than in Western Europe and North America, and most areas were not covered by large ice sheets during the late Pleistocene^2^, in addition, East Asia is a mountainous mosaic area (Fig. 1) and has the potential to host microclimatic zones that are probably capable of supporting a variety of habitats in relative stability^4^,^5^.

Biological invasions and climate change are considered to represent major threats to biodiversity, ecosystem functioning and agroforestry^6^. Many factors influence the establishment of a non-indigenous pest into a community (e.g., wide physiological tolerance, generalist behavior in host choice, or favorite biotic interaction). Comparing to other specialist or endemic species, invasive pests may not be seriously affected by Pleistocene climate because of their wide ecological flexibility or high dispersal ability^1^. Revealing their response to Pleistocene climate provides useful information on their responding to the ongoing global climate change^1^,^7^. At present, the phylogeography to track species’ responses to climate change have put much emphasis on the none-invasive species; studies into the history and /or future of invasive species are scant.

Recently, ecological niche modelling (ENM) emerged as an important tool in the studies of biological invasion and phylogeography^8^. The general idea behind ENM is to characterize a model of species’ realized niche. Based on niche conservatism^9^, the characterized niche can be projected to identify areas of potential distribution. A problem with ENM approach in generating invasive species’ potential distribution is that this correlative approach predicts species’ distributions without explicitly incorporating processes that potentially limit geographic distribution^10^. To more accurately predict invasive species’ distributions, consideration needs to be given to the invasive ability (e.g., dispersability, adaptability, and demography). The characterized niche can also be projected to identify areas of historical distribution^11^. This approach to generate historical distribution is spatially explicit and independent from hypotheses built upon phylogeography^12^. Although many sources of uncertainty existing in ENM (e.g., low niche model transferability) and phylogeography (e.g., population divergence estimation), a coupled phylogeographical and ENM approach could strengthen the hindcasting of species’ historical distributions^12^,^13^,^14^.

The Brown Marmorated Stink Bug (BMSB), *Halyomorpha halys* (Stål) (Hemiptera: Pentatomidae), native to East Asia, has become an invasive species in the US and Europe^15^. In East Asia, the species spans from temperate to subtropical zones, feeding on a wide variety of fruit and ornamental trees. In the US, BMSB was accidentally imported in the late 1990s, the invader recently emerging as a key pest in agriculture, creating major nuisance problems especially in the mid-Atlantic region. In Europe, it was first discovered in Switzerland in 2008 and was later found in Liechtenstein, Germany and France. The northern China populations have been proved to be the original sources of BMSB invasion in the US^16^, and also likely to be the source of European invasion in Switzerland^17^.

In this study, we first test climate niche conservatism across BMSB geographic populations and monophyletic clades, we then linked phylogeography and paleoclimate niche modelling to derive information on BMSB population in late Pleistocene. Here we use rangewide mitochondrial markers coupling with nuclear data to investigate the phylogeographic structure of BMSB, and to explore its demographic changes in response to Pleistocene fluctuations using coalescent-based methods. With lessons from the past, we recalibrate the niche model for BMSB to assess its climatic favorable zone under future climate scenarios in the end. The aims of this study are to (1) reveal the phylogeographic structure of BMSB in East Asia, (2) to explore their demographic response to Pleistocene climatic fluctuations, and (3) to predict the effect of future climatic change on the potential distribution of BMSB.

## Results

### Genetic diversity and phylogeographic structure

2443 bp of protein-coding regions in mitochondrial genome were obtained (COI: 1349 bp; CYTB: 1094 bp) from 234 specimen. 183 unique haplotypes were derived. The 275 polymorphic sites included 100 singleton variable sites and 175 parsimony informative sites. Nucleotide diversities for mtDNA ranged from 0.00018 to 0.00563 (Supplementary Table S1), with an average of 0.00725. High haplotype diversity was observed in the entire sample, ranging from 0.275 to 1.000 with an average of 0.9869 (Supplementary Table S1). For the nuclear sequencing data, 416 bp ITS1 fragment was successfully obtained from 40 individuals, covering regions of mainland China, Korea and Taiwan island. 8 unique haplotypes were derived. The 10 polymorphic sites included 1 singleton variable sites and 9 parsimony informative sites.

**Figure 1:**
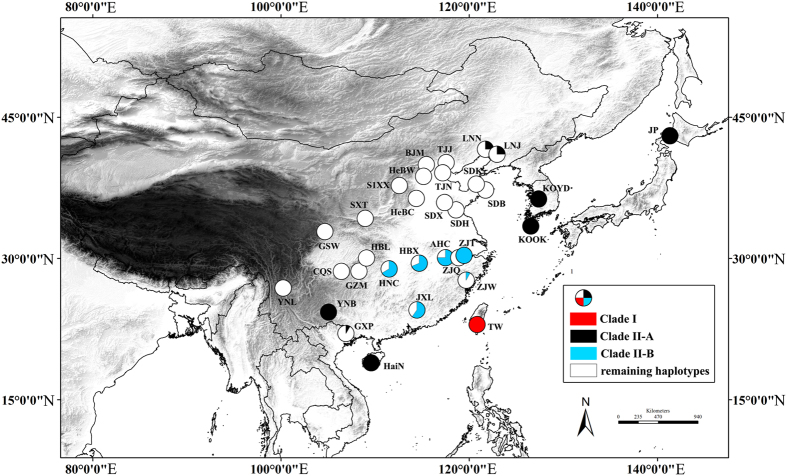
Geographic distribution of three monophyletic clades in East Asia. Haplotypes frequencies of clade I, clade II-A and clade II-B were shown by the pie graph. Figure was generated in ArcGIS 10 (Environmental Systems Research Institute).

**Figure 2:**
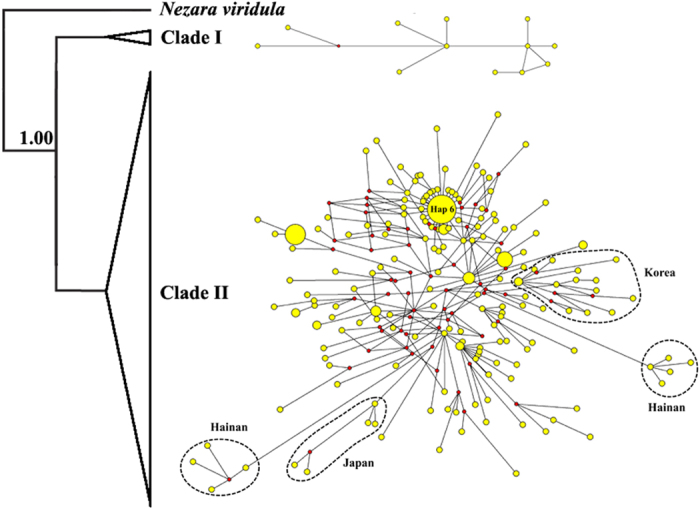
Mitochondrial DNA tree and the nested clade networks using combined COI and Cytb sequences. Bayesian support values are marked up the branches.

**Figure 3:**
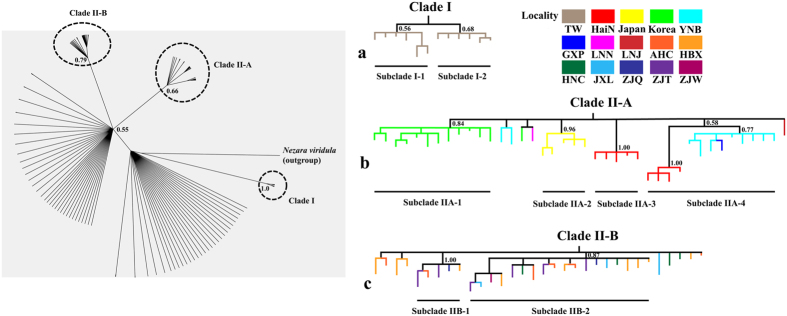
Three monophyletic clades in Bayesian phylogenetic tree based on the combined COI and CYTB sequences. Details and distributions of haplotypes within the three clades are shown in the right panel. Bootstrap values are indicated at each node.

Bayesian inference (BI) analysis suggests that the native BMSB populations could be separated into two clades (Fig. 2), clade I (Taiwan clade) and II (mainland China, Korea, and Japan). Two additional monophyletic subclades (clade II-A and clade II-B) were recognized in clade II (Fig. 3). Geographical distributions of the three monophyletic clades were uneven (Fig. 1). The clade I was solely found in Taiwan (i.e., TW); whereas clade II-A was common in Korea (i.e., KOOK and KOYD), Japan (i.e., JP), Hainan (i.e., HaiN), and eastern Yunnan populations (i.e., YNB), with a few found in Liaoning (i.e., LNN and LNJ) and Guangxi populations (i.e., GXP), and clade II-B occurred mainly along the Yangtze River (i.e., AHC, HBX, HNC, ZJQ, ZJT and ZJW). The discontinuous distribution pattern of clade II-A might be on account of long-distance dispersal itself or human-mediated transportation. Two independent networks (i.e., Taiwan clade, and mainland China, Korea and Japan clade) were identified in the network analysis, suggesting a long-term isolation. Within clade II, the mainland China network was complex, with high level of reticulation (Fig. 2). The network overall has a multiple star-like shape, a typical character of population expansion^18^. For the nuclear data, the network analysis also showed that Taiwan population was diverged from the other native populations, which consolidated the result of mitochondrial data (Supplementary Fig. S1).

### Demographic history on mitochondrial data

The potential area for each ancestral node within three monophyletic clades was identified (i.e., clade I, clade II-A and clade II-B). Assignment of ancestral areas indicates that clade I was originated from Taiwan (Fig. 3). The haplotypes of subclade IIA-1, IIA-2 and IIA-3 were directly attached to clade II-A, they were from Korea, Japan and Hainan respectively, suggesting these areas (HaiN, KOOK, KOYD and JP) might be the origins for BMSB expansion, whereas subclade IIA-4 was composed of multiple populations (i.e., Hainan, Yunnan and Guangxi). The basal node of clade II-B was not well resolved (Fig. 3).

**Figure 4:**
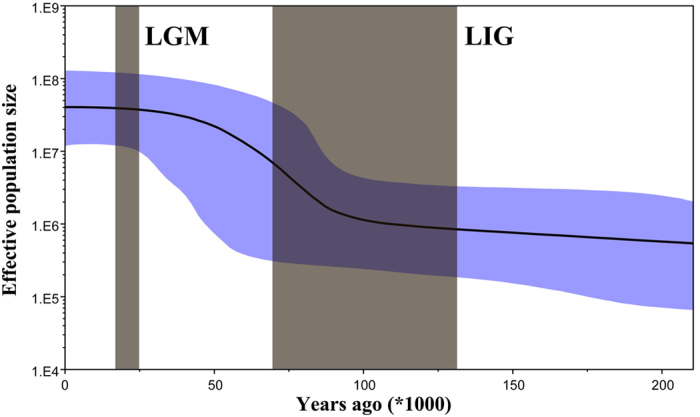
Historical demographic trends represented by Bayesian skyline plot (BSP) for BMSB. Solid line indicates the mean of effective population size, and the shaded range delineates the 95% confidence interval.

Divergence time between Clade I (Taiwan) and Clade II (mainland China, Korea and Japan) was estimated to be 0.79 million years (MY) ago (95% HPD: 0.36–1.00 MY ago), which appeared in the mid–Pleistocene (Supplementary Fig. S2). In the later clade, little phylogeographic structure was observed (Fig. 2). The mismatch distributions for Clade II and entire sample were a distinct unimodal curve (Supplementary Fig. S3), suggesting BMSB populations did not significantly differ from a model of population expansion; this expansion scenario was supported by the negative values of Tajima’s *D*, Fu and Li’s *D* and Fu’s *F*_S_, and the larger than expected number of singleton mutations (Table 1). We then used the entire COI data except Taiwan to estimate population expansion time. From *t *= *τ*/2*u*, we estimated the expansion time ranged from approximately 55000–92000 years ago. Using BSP (Bayesian Skyline Plot), the historical population dynamics were visually displayed (Fig. 4); the demographic trend indicates the advent of a phase of rapid demographic growth after a prolonged phase of substantial demographic stability, which started about 88000 years ago (Fig. 4).

### Principle component analysis

**Figure 5:**
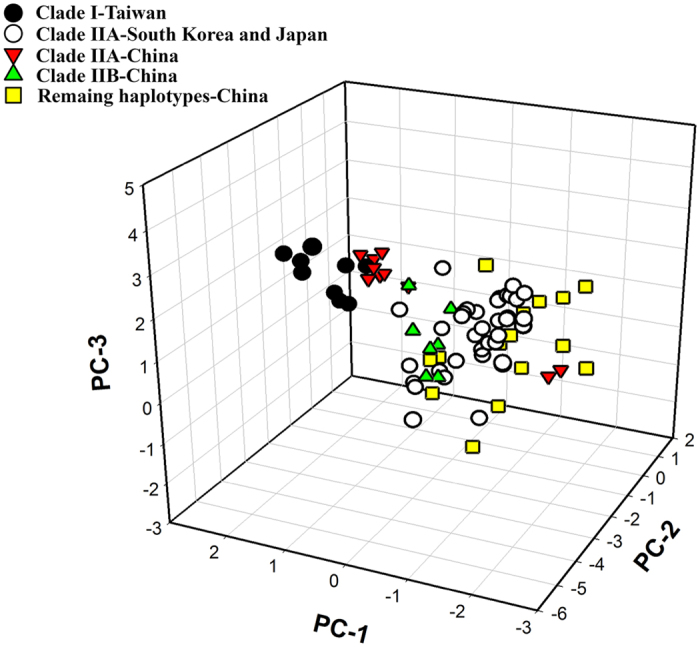
Principle component analysis of five bioclimate variables associated with occurrence of BMSB in native East Asia. Symbols represent monophyletic clades occurred in Taiwan, mainland China, South Korea, and Japan.

Principle component analysis of five bioclimatic variables associated with BMSB occurrence revealed reduced dimensions that account for the observed distribution (Fig. 5, Supplementary Table S3). The first three components were significant, together explaining 94.78% of the variance. The first component (PC-1) was associated with the annual mean temperature and minimum temperature of the coldest month; the second (PC-2) was associated with the maximum temperature of the warmest month; while the third (PC-3) was less clearly associated with a single dimension (Supplementary Table S2). The clade IIA of South Korea and Japanese populations were all clustered within a cloud of Chinese occurrences, suggesting climate niche conservatism among these populations and clades, whereas the Clade I Taiwan population showed somewhat departing from the mainland cloud (Fig. 5) along the first axis which was associated with the annual mean temperature and minimum temperature of the coldest month (Supplementary Table S2).

### Ecological niche modeling

**Figure 6:**
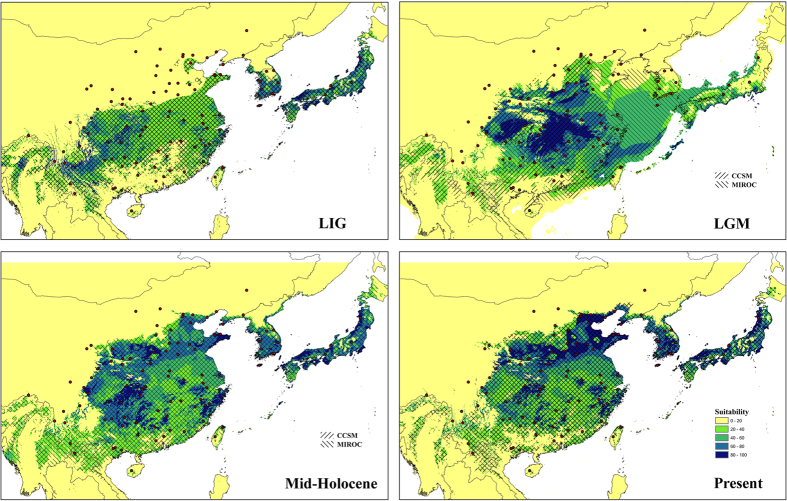
Hindcasting the current BMSB niche model onto the LIG (~75000–125000 years ago), LGM (~21,000 years ago) and Mid-Holocene (~6000 years ago) period using Maxent. Niche model results were modified in ArcGIS 10 (Environmental Systems Research Institute). Red dots indicate occurrence records used to fit niche model. Slash areas suggest model predictions approved by the 10th training presence threshold.

Niche models based on the native climate space showed good transferability in capturing the introduced records in Europe and the US (*X*^2^ = 106.89; df = 1, *p *< 0.05), suggesting climate niche stability across geographic space. When projecting the current niche into historical climate conditions, a range-expanding trend was observed throughout the LIG (Last Interglacial, ~75000–125000 years ago), LGM (~21,000 years ago), and Mid-Holocene (~6000 years ago) periods. In binary predictions, during the LIG period, the suitable climate space is distributed in central and southwestern China, and most parts of Japan and South Korea. When the ice age arrived (LGM period), the suitable space in Japan and southwestern China vanished, whereas the central space in China showed expansion toward both the north and south; an eastern expansion was also observed in the land bridges that connected China and Japan during the LGM. When ice retreated, the suitable space continued its northward expansion, occupying most of Japan and Korea, forming the current BMSB distribution in East Asia (Fig. 6). In probability predictions, high suitable space was observed in southwestern China, and in most parts of South Korea and Japan during LIG; the suitable space in central China increased when the ice age started. When ice retreated, the high suitable space shifted northward in China, South Korea and Japan (Fig. 6).

**Figure 7:**
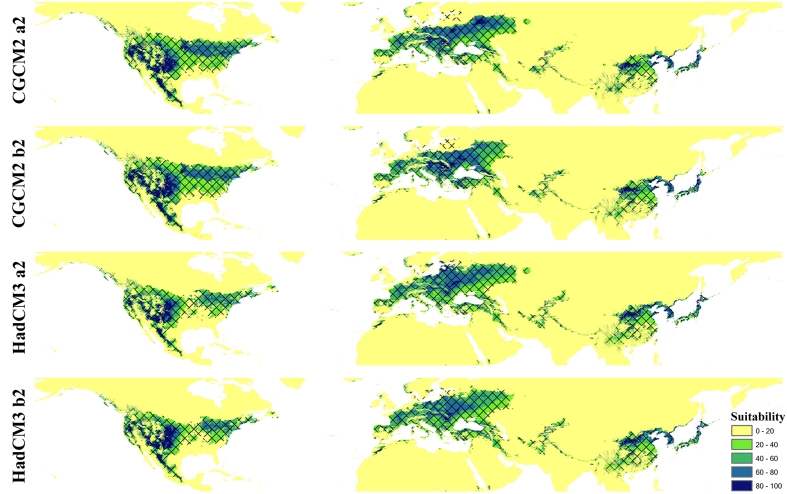
Future projections of BMSB invasion potential in 2050 under different climate scenarios using Maxent. Niche model results were modified in ArcGIS 10 (Environmental Systems Research Institute). Slash areas suggest model predictions approved by the 10th training presence threshold.

Global transferring of the native current niche model into future climate conditions showed different degrees of southern contraction and northern expansion in the US. A northeastward expanded suitable space was observed in Europe. Reduced suitable space was noticed in central China (Fig. 7). A similar pattern was observed, but to a lesser degree, in the global-based model projection of invasion potential (Supplementary Fig. S4).

## Discussion

### Little phylogeographic structure in Clade II

Limitations on the material and methodology employed in this study need to be addressed here. Examining phylogeography and evolutionary history solely based on mtDNA sequences involves a significant risk of misinterpretation^19^. Comparing with the former study^16^,^17^, our mtDNA data covered the entire geographic extant of BMSB in its native range and used more large datset, in addition, the results of which were also consolidated by the nuclear data and the spatial niche model predictions. Both molecular clock and Yule tree suggest that the divergence between clade I (Taiwan) and clade II (mainland China, Korea and Japan) occurred around 0.79 (0.36–1.00) million years ago (Supplementary Fig. S2). Our planet experienced a dramatic climate shift during this period, which termed “mid-Pleistocene revolution”^20^. The relatively disjuncted positions between clade I and clade II in climate space suggested that niche space of Taiwan population might be diverged from mainland China, Korea and Japan (Fig. 5). It is widely accepted that ecology plays an important role in speciation^21^,^22^. Therefore it is possible that the drier and colder environment in the mid-Pleistocene climate transition that have facilitated the genetic divergence of clade I and II.

In mainland China, Japan and Korea populations, the genetic exchange tends to occur among the surrounding areas (Fig. 2), this might be happened through the natural dispersal and/or temporary mating dispersal^23^. Multi-reticulations in the haplotypes network gave a complex topology that was not easily resolvable (Fig. 2), which points to the recurrent and abundant gene flow among populations^24^. Little phylogeographic structure in mainland China populations suggests that a prolonged allopatric divergence might not have occurred. In haplotype network, the long-branch was observed in Hainan and Japan populations (Fig. 2), whereas the short branch and starlike existed in the remaining mainland China (i.e., north and central China) populations, suggesting the mainland populations have experienced expansion while the Hainan and Japan populations might be maintained stable^25^. The geographic barrier of the strait in Hainan and Japan might restrict gene flow among populations.

### Colonization of northern China populations after LIG

The northern China populations have been proved likely to be the original sources of BMSB invasion in the US^16^ and Europe^17^. Result of BSP showed rapid demographic growth from LIG to LGM (Fig. 4). This was consistent with the spatial historical prediction of ENM (Fig. 6). This result disagrees with the demographic expansion time of posterior LGM (i.e., 6000–15000 years ago), which is based on a mutation rate 6.2%/per million years (an estimation for *Drosophila* mtDNA^16^). Comparing ENM predictions between LIG and LGM, a great expansion in suitable space was observed in northern China. The haplotypes in clade I, clade IIA and clade IIB might not be the sources for northward colonization because of their high genetic divergence (Fig. 1). The network of northern China populations, the widespread haplotype Hap 6 and many its newly derived haplotypes comprised the core (Fig. 2). This Hap 6 was also found in the central China populations (e.g., CQS, YNL and ZJW), we speculated that that the Hap 6 originated in central China and colonized to the north. After that, it derived many new haplotypes to adapt the novel environment (Fig. 2). High genetic diversity and multi-reticulations (Fig. 2, Supplementary Table S1) suggest the northern China populations possess high dispersability and/or adaptability^26^.

### Historical demography

Combined molecular phylogeography and ecological niche modelling revealed an unusual rapid pre-LGM expansion after a prolonged phase of demographic stability (Figs 4 and 6). Many factors may contribute to the persistence of interconnected populations rather than population contraction into separate glacial refugia. First, BMSB exhibited high ecological flexibility in the field. BMSB is highly polyphagous, with >300 reported host plants^27^,^28^. The host species exist in a wide range of plant families. Some of these plants might persist through LGM in northern China^29^,^30^,^31^, or China mountain areas^32^,^33^,^34^; they could have served as food source for BMSB during LGM. In addition, the milder Pleistocene climate in East Asia might also have mitigated demographic stresses on BMSB. During glacial periods, the ice-sheets did not cover most of China, and the glacial cycles effects were less dramatic compared to Europe and North America. Most mountains in southeastern Asia have the potential to host microclimatic zones^5^,^32^,^33^. Therefore BMSB populations could have survived during the climatic oscillations, and exploited large areas of potentially suitable environmental conditions throughout late Pleistocene.

### Future projections

Under future climate scenarios, the introduced BMSB populations therefore likely expand northward to higher latitudes, invading previously unaffected regions in the US and Europe. The difference between native- and global-range based models in future projections might be due to the effect of geographic background and population states^35^. Our prediction was consistent with the observed BMSB population in the US. Recently, BMSB adults were continuously intercepted in the state of Florida from the highly suitable area in the central Atlantic US states; however, nymphs and eggs have never been found, suggesting BMSB haven’t established populations in Florida (Julieta Brambila, USDA-APHIS, per. com.), which is most likely due to the unsuitable climate in southeastern US.

## Conclusion

Phylogeography coupled with ENM revealed the Pleistocene history of the invasive species BMSB in the native range. Mitochondrial and nuclear data both revealed significantly genetic differentiation between the populations from Taiwan and the other native populations. Divergence time approximately coincided with the period of “mid-Pleistocene revolution”, which indicated that the drier and colder environment in this period might facilitate the genetic divergence. Combined BSP and niche modelling revealed the native BMSB persisting in interconnected populations and undergo a rapid expansion during the transition of LIG to LGM. High genetic diversity and multi-reticular haplotypes network existed in the original sources populations of BMSB invasion in northern China, which were probably colonized from the central China. The ENM future prediction suggested BMSB may expand northward to higher latitudes in the US and Europe under the future climate conditions. The wide ecological flexibility and relatively high dispersal ability, together with the milder Pleistocene climate in East Asia, could have shaped the demographic history of this invasive species in the native area. We provided a successful example of uncovering the genetic structure and underlying factors of a global invader in the native range. Further BMSB studies are needed to obtain more molecular data from invasion areas (e.g. US and Europe) and using larger nuclear dataset (e.g. SNP) to reveal genetic structure and demographic history in the global process of biological invasion.

## Methods

### Sampling and sequencing

All the experimental protocols and procedures involving *H. halys* were approved by the Committee for Animal Experimentation of the College of Life Science at Nankai University (no. 2008) and were carried out in accordance with the approved guidelines. Tissue samples were obtained from a total of 234 individuals corresponding to 32 different populations across the native rangewide of BMSB in East Asia (Fig. 1, Supplementary Table S1). In each location, 2 to 16 individuals were captured and preserved in 95% ethanol in the field, and then stored in a freezer (−40 °C) until DNA extraction. Total genomic DNA was extracted from the thorax tissue and isolated using either a CTAB-based method^36^ or a General AllGen Kit. Polymerase chain reactions (PCRs) were performed using specific primers designed in the present study (Supplementary Table S2). The PCR procedure for COI, CYTB and ITS1 included an initial denaturation at 94 °C for 2 minutes, followed by 31–33 cycles of 30 seconds at 92 °C, 30 seconds at 51–52 °C and 1–1.25 minutes at 72 °C, ending with a final extension at 72 °C for 8 minutes. All fragments were sequenced in both directions using a HiSeq 2000 sequencing system. Sequences were visually proofread and aligned in Bioedit 7.1^37^.

### Genetic analysis

Genetic diversity was estimated for each location and entire sample as the number of polymorphic sites (*S*), number of haplotypes (Nhap), haplotype diversity (*Hd*), and nucleotide diversity (*π*), which were calculated in DNASP 4.0^38^.

Bayesian inference (BI) was used to construct the phylogenetic tree. Model test (version 3.7) was then used to choose the appropriate substitution model and TIM+I+G was selected as the best. The number of generations was 15 million, with tree sampled every 1000 generations until the average standard deviation of split frequencies was below 0.01; the first 25% of generations was discarded. Genealogical relationships between haplotypes were investigated by constructing a phylogenetic network using Median-Joining model implemented in Network 4.6.1.1 software (Fluxus Technology Ltd.). Haplotype network can be used to infer population history, as the most ancient haplotypes should be located at the center of gene tree and be geographically widespread, whereas the most recent haplotypes should be at the tips and be localized geographically^39^. The ancestral haplotypes are likely to be found at locations of expansion origins whereas the derived haplotypes likely found in newly recolonized regions. We used the maximum parsimony principle to reconstruct the possible location of each ancestral node for major clades on the inferred phylogenetic tree^40^.

Mismatch distribution was used to explore the native demographic history of BMSB. Multimodal mismatch distributions are assumed to characterize old populations of constant size whereas expanding populations tend to be unimodal^41^. Here mismatch distribution for all BMSB individuals were calculated with the expected frequency based on a population growth-decline model in DNASP 4.0^38^. The ruggedness index (*r*) can be used to quantify the smoothness of mismatch distribution^41^. Under a population growth model, the *r* values are expected to be low and can be tested for deviation from the constant population model. In addition, three neutrality tests, i.e., Tajima’s *D*^42^ and Fu and Li’s *D*^43^ calculated in DNASP and Fu’s *F*_S_ in Arlequin 3.5^44^ were used to detect departures from the mutation-drift equilibrium. The significant negative values of Tajima’s *D*, Fu and Li’s *D* and Fu’s *F*_S_ tests would be interpreted as signatures of historical population expansion.

We used two methods to estimate population expansion time based on COI sequence data. One approach is using *t *= *τ*/2*u* (*t* is the expansion time, *τ* is the crest of mismatch distribution, and *u* is the mutation rate per generation); the value *u* was calculated using the formula *u *= *μk* (*μ* is the mutation rate per nucleotide and *k* is the number of nucleotides assayed). Referring to the molecular evolution in assassin bug (Hemiptera: Reduviidae)^45^, we assumed a mutation rate 0.6%–1%/per million years, and adopted a scheme of two generations for native BMSB. The other approach is using a Bayesian coalescent-based method (Bayesian Skyline Plot, BSP); this method was used to estimate the time of the most recent common ancestor (TMRCA) and detailed demographic history data back to TMRCA^46^. The chains were run for 100 million generations until the effective sample sizes were more than 200, and the first 10% were discarded. The flexible mode (i.e., GTR+G+I substitution model)^47^ and a relaxed uncorrelated lognormal molecular clock with the mutation rate of 0.6%–1%/per million years for BMSB were used^45^. Demographic history was reconstructed in Tracer 1.4. Divergence time was estimated in a Bayesian framework based on a coalescent method. Here the analysis was performed using a Yule Process model, with the chains run for 50 million generations sampling every 1000 generations. TreeAnnotator 1.6.1 (in BEAST package) in the end was used to summarize trees with “Mean height”, and discarded the first 25%.

### Ecological niche modeling

Insects are poikilothermic organisms and all aspects of their biology depend on the effective temperature^48^. Other nonclimatic factors, like bionomics and occupancy dynamics, might also operate but rather at a local scale^49^. The use of annual mean and extreme climate conditions data have proven to be effective as both climate features have a significant effect on insect population and distribution^50^. We used five bioclimatic variables^51^ that are thought to most likely restrict BMSB distribution^15^, i.e., annual mean temperature (BIO1), mean diurnal temperature range (BIO2), maximum temperature of the warmest month (BIO5), minimum temperature of the coldest month (BIO6), and annual mean precipitation (BIO12). All the variables were derived from the WorldClim data center at a resolution of 5-arc. Prior to niche modeling, the principle component analysis was performed to identify the suitable environmental variables in reduced dimensions among the geographically or monophyletic separated populations. Principal component analysis provided a representation of the species’ climate space across populations. The clustering of occurrences suggests climate niche conservatism while separation signifies a potential divergence from niche conservation, in a strict sense^52^.

Maximum entropy that implanted in Maxent^53^ was used to estimate niches in environmental dimensions. Analysis was run on default program conditions (cumulative output, convergence threshold (10^−5^), maximum number of iterations (500)). To hindcasting the Pleistocene climate effect, we adopted the 95 occurrence records in Zhu *et al.*^15^ for niche modeling. These records were evenly distributed across the geographic space, which were screened from 383 native records in order to reduce sample bias and spatial autocorrelation. The current native niche models were calibrated and projected onto the reconstructed climatic conditions during LIG, LGM and Mid-Holocene periods simulated by Community Climate System Model^54^. Two general circulation models (GCM) for Mid-Holocene and LGM condition were used, the Community Climate System Model (CCSM) and the Model for Interdisciplinary Research on Climate (MIROC). Model predictions based either on CCSM or MIROC were summed as the consensus for LGM prediction. The future projections were based on a consensus-forecast approach that combined the CGCM2 and HADCM3 climate models under two emission scenarios (a2 and b2) by 2050. These climate scenarios drawn from the Hadley and Canadian climate modeling centers, in each case for the a2 and b2 emissions scenarios, are most likely to bracket the range of future climate conditions (i.e., being relatively liberal and relatively conservative). Niche models were calibrated on both native range and whole world for future projection^55^. 198 records screened from the global 552 records (see protocol in Zhu *et al.*^15^) were used for the global-based model projection.

The choice of threshold used to derive binary predictions altered projections of species range under current, historical and future climate conditions because any fixed threshold to transform model output will mask the possibility prediction and exaggerate prediction errors^56^. We therefore overlaid probability prediction with binary prediction to exhibit predictions that were masked by the latter. To do this, we firstly thresholded model predictions to produce binary maps by establishing the level at which 90% of the input occurrence points were included in the prediction. To test the authenticity of climate niche transferring across temporal space (i.e., historical and future climate conditions), we tested native niche model transferability across the geographic space by their ability to capture the introduced occurrence points in the US and Europe. Statistical significance was tested by comparing the proportional area predicted as occupied by BMSB against the number of test points that would be occupied if BMSB were distributed randomly.

## Additional Information

**How to cite this article**: Zhu, G.-P. *et al.* Range wide molecular data and niche modeling revealed the Pleistocene history of a global invader (*Halyomorpha halys*). *Sci. Rep.* **6**, 23192; doi: 10.1038/srep23192 (2016).

## Supplementary Material

Supplementary Information

## Figures and Tables

**Table 1:** Nucleotide polymorphism and Neutrality tests in defined groups and whole data.

Parameter	Taiwan	mainland China,Korea, and Japan	Whole set
Sample size	12	222	234
*S*	20	221	275
Nhap	12	171	183
*Hd*	1.000	0.9854	0.9869
*π*	0.00220	0.00371	0.00725
Tajima’s *D*	−0.82507	−2.39457**	−1.90645*
Fu’s Fs	−7.78413	−24.31987***	−23.71521***
Fu and Li’s D*	−1.11138	−5.72864**	−3.89412**

*S*, number of segregating sites; NHap, number of haplotypes; *Hd*, haplotype diversity; *π*, nucleotide diversity.

*p < 0.05.

**p < 0.02.

***p < 0.001.
