# Supplementary material for: Range wide molecular data and niche modeling revealed the Pleistocene history of a global invader (Halyomorpha halys)
Source: Sci Rep. 2016 Mar 21;6:23192. doi: 10.1038/srep23192 (PMC4800403; doi:10.1038/srep23192)

**Range wide molecular data and niche modeling revealed the** **Pleistocene history of a global invader (*Halyomorpha halys*)**

Geng-Ping Zhu^1^,^2^,*, Zhen Ye^2^,*, Juan Du^2^,*, Dan-Li Zhang^2^, Ya-hui Zhen^2^, Chen-guang Zheng^2^, Li Zhao^1^, Min Li^1^ & Wen-Jun Bu^2^

^1^Tianjin Key Laboratory of Animal and Plant Resistance, College of Life Sciences, Tianjin Normal University, 393 Binshui Road, Tianjin 300387, China, ^2^College of Life Sciences, Nankai University, 94 Weijin Road, Tianjin, 300071, China.

Corresponding

Wenjun Bu

College of Life Sciences, Nankai University, 94 Weijin Road, Tianjin, 300071, China.

[wenjunbu@nankai.edu.cn](mailto:wenjunbu@nankai.edu.cn);

Running title: Pleistocene history of *H. halys*

Manuscript type: Original Article

**Supplementary information**

**Table S1** **|** Nucleotide polymorphism in each population with geographical coordinates. *S*, number of segregating sites; Nhap, number of haplotypes; *Hd*, haplotype diversity; *π*, nucleotide diversity.

| CYTB+COI | Latitude | Longitude | Sample size | *S* | Nhap | *Hd* | *π* |
| --- | --- | --- | --- | --- | --- | --- | --- |
| AHC | 30°5'24"N | 117°28'48"E | 8 | 20 | 8 | 1.000 | 0.00253 |
| BJM | 39°58'48"N | 115°26'24"E | 9 | 16 | 6 | 0.833 | 0.00171 |
| GSW | 32°52'12"N | 104°39'36"E | 5 | 21 | 5 | 1.000 | 0.00385 |
| GXP | 22°3'36"N | 106°53'24"E | 12 | 28 | 12 | 1.000 | 0.00226 |
| GZM | 28°39'36"N | 108°16'48"E | 8 | 18 | 8 | 1.000 | 0.00212 |
| HaiN | 18°55'12"N | 109°35'24"E | 9 | 29 | 9 | 1.000 | 0.00563 |
| HeBC | 36°23'24"N | 114°22'12"E | 5 | 7 | 5 | 1.000 | 0.00131 |
| HeBW | 38°44'24"N | 115°9'2"E | 3 | 3 | 3 | 1.000 | 0.00082 |
| HBL | 30°3'36"N | 109°4'48"E | 3 | 9 | 3 | 1.000 | 0.00246 |
| HBX | 29°31'12"N | 114°41'24"E | 16 | 44 | 15 | 0.992 | 0.00301 |
| HNC | 28°54'36"N | 111°29'24"E | 6 | 17 | 6 | 1.000 | 0.00265 |
| JXL | 24°33'2"N | 114°27'1"E | 5 | 25 | 5 | 1.000 | 0.00458 |
| LNJ | 41°36'36"N | 121°43'12"E | 4 | 18 | 4 | 1.000 | 0.00389 |
| LNN | 41°5'24"N | 122°57'36"E | 4 | 14 | 3 | 0.833 | 0.00300 |
| SDB | 37°16'48"N | 121°44'24"E | 6 | 8 | 5 | 0.933 | 0.00156 |
| SDH | 35°10'12"N | 118°36'36"E | 2 | 6 | 2 | 1.000 | 0.00246 |
| SDK | 37°54'36"N | 120°44'24"E | 2 | 1 | 2 | 1.000 | 0.00041 |
| SDX | 35°57'1"N | 117°22'48"E | 10 | 11 | 4 | 0.733 | 0.00148 |
| S1XX | 37°45'36"N | 112°34'12"E | 14 | 3 | 3 | 0.275 | 0.00018 |
| SXT | 34°16'12"N | 108°57'1"E | 8 | 12 | 5 | 0.857 | 0.00152 |
| TW | 23°1'36"N | 120°50'24"E | 12 | 20 | 12 | 1.000 | 0.00220 |
| TJJ | 40°11'24"N | 117°32'24"E | 5 | 15 | 5 | 1.000 | 0.00254 |
| TJN | 39°6'36"N | 117°10'12"E | 11 | 8 | 6 | 0.727 | 0.00065 |
| YNB | 24°19'12"N | 105°2'24"E | 10 | 23 | 10 | 1.000 | 0.00226 |
| YNL | 26°52'12"N | 100°13'48"E | 2 | 1 | 2 | 1.000 | 0.00041 |
| ZJQ | 30°5'24"N | 118°52'48"E | 5 | 15 | 5 | 1.000 | 0.00287 |
| ZJT | 30°21'5"N | 119°27'1"E | 6 | 28 | 6 | 1.000 | 0.00415 |
| ZJW | 27°42'36"N | 119°40'48"E | 13 | 27 | 12 | 0.987 | 0.00207 |
| CQS | 28°38'60"N | 106°24'36"E | 12 | 24 | 11 | 0.985 | 0.00220 |
| JP | 43°2'31"N | 141°18'59"E | 5 | 8 | 5 | 1.000 | 0.00164 |
| KOOK | 33°26'59"N | 126°31'36"E | 4 | 10 | 4 | 1.000 | 0.00205 |
| KOYD | 36°19'54"N | 127°21'32"E | 10 | 15 | 10 | 1.000 | 0.00162 |

**Table S2 | Primer sequences that were used to amplify the two mitochondrial fragments used in this study.**

| **Gene region** | **Primer name** | **Sequence (5′–3′)** |
| --- | --- | --- |
| **COI** | gb1f | ATTAGGACAGCCTGGAAG |
|  | gb1r | CATAATGGAAATGGGCTACTACA |
|  | 1914ZJF | GTATAACCCCTGAACGAATCCCAT |
|  | 3005NVR | TCCTCATGTTGCTATTTCTA |
| **CYTB** | 10270gb1f | TTTTGGACCTTTACGAAT |
|  | 11645gb1r | AGGAGTTTTACCCCGATT |
| **ITS1** | L1 | CGTAACAAGGTTTCCGTAGG |
|  | R1 | CAGTTTGCTGCGTCTTTCAT |

**Table S3 |** Principal component analysis (PCA) of bioclimatic variables associated with native BMSB occurrence. Eigenvalues for the most important variables (> 0.85) in PCA are in bold.

| **Variables** | **Description** | **Factor Loadings** | | |
| --- | --- | --- | --- | --- |
|  |  | PC-1 | PC-2 | PC-3 |
| **BIO1** | Annual mean temperature | **0.91** | 0.34 | 0.15 |
| **BIO2** | Mean diurnal temperature range | -0.78 | 0.40 | 0.49 |
| **BIO5** | Maximum temperature of warmest month | 0.37 | **0.88** | -0.14 |
| **BIO6** | Minimum temperature of coldest month | **0.96** | 0.02 | 0.10 |
| **BIO12** | Annual precipitation | 0.78 | -0.45 | 0.26 |
| **Eigenvalue** |  | 3.12 | 1.26 | 0.36 |
| **Percentage variance** | | 62.35 | 25.17 | 7.25 |
| **Cumulative percentage variance** | | 62.35 | 87.53 | 94.78 |

**Figure S1 |** Median joining haplotype network of nuclear data constructed using Network. Haplotype circle size denotes the number of sampled individuals. Numbers of base pair changes (no number = 1 bp) are given.


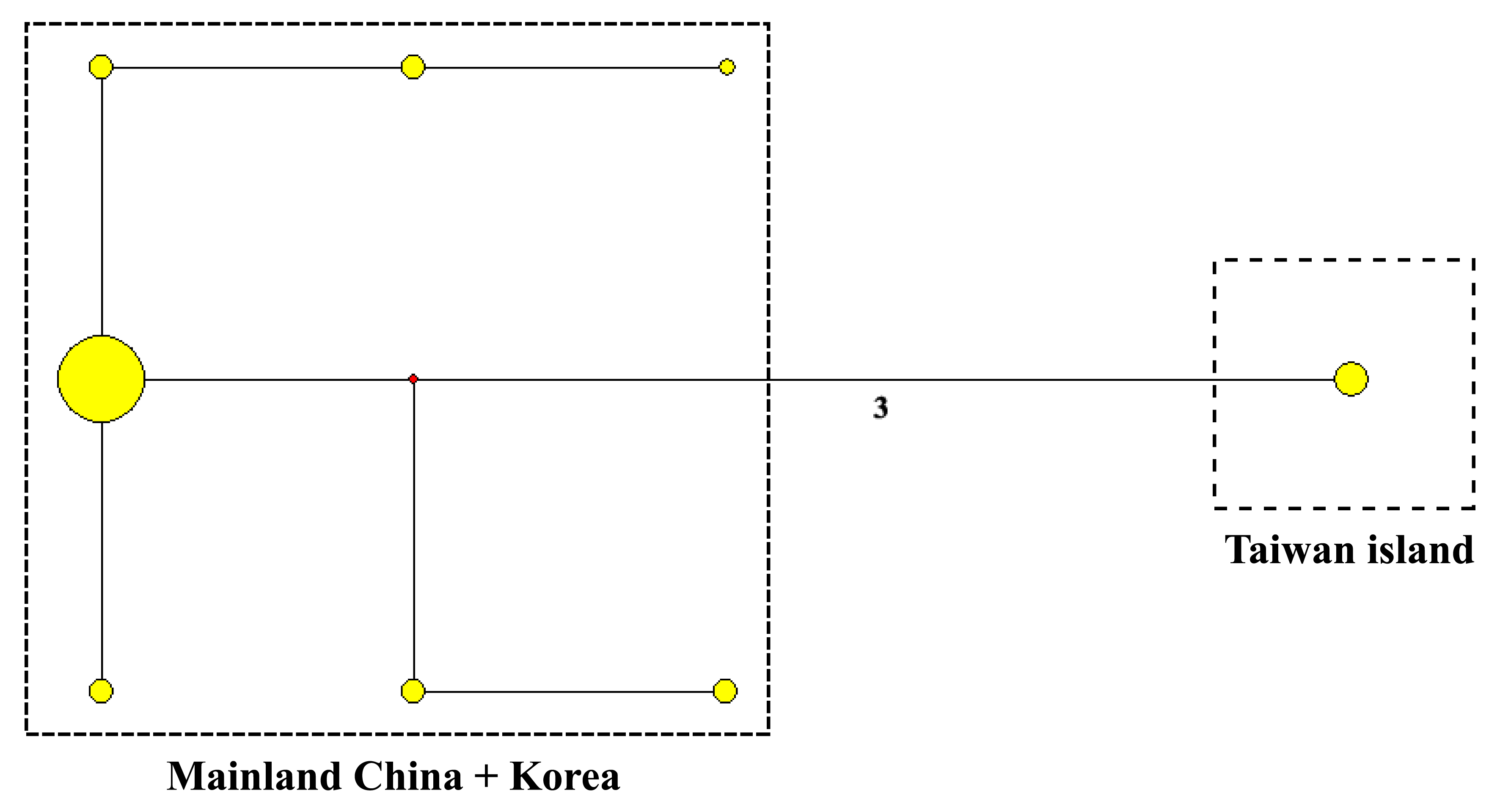


**Figure S2 |** Yule tree and divergence time between clade I and II constructed using COI sequences for BMSB**.**

**
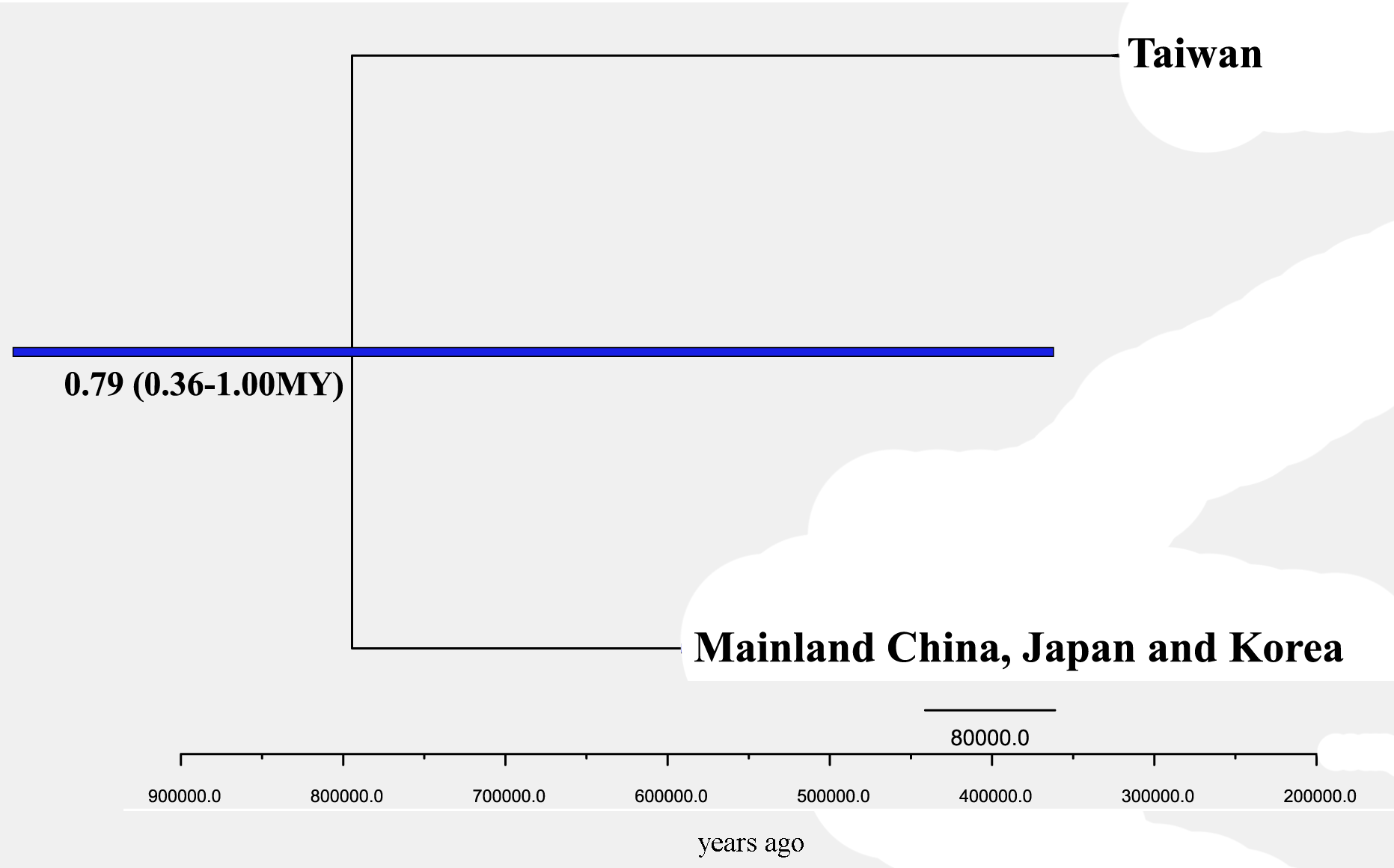
**

**Figure S3 |** Mismatch distributions for the Clade I (a), Clade II (b) and the entire sample (c). Curves represent the frequency of observed (blue dots line) and expected (red continuous line) distribution of pairwise differences. P values indicate whether pairwise distributions are significantly different from the stepwise expansion model.

**
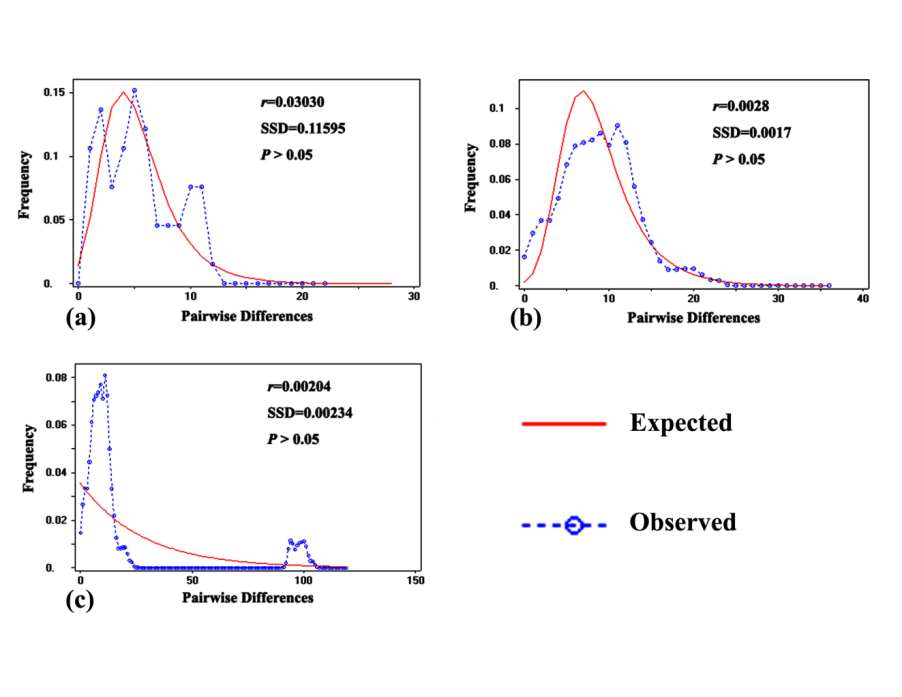
**

**Figure S4** | Future projections of BMSB invasion potential in 2050 under different climate scenarios using Maxent. Niche model results were modified in ArcGIS 10 (Environmental Systems Research Institute). Slash areas suggest model predictions approved by the 10th training presence threshold.


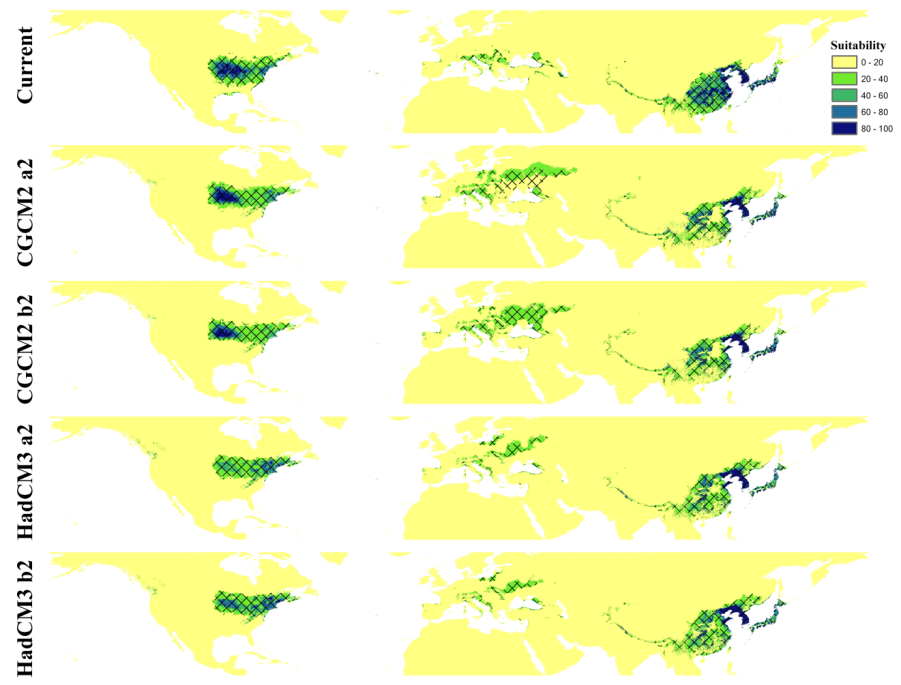

Supplement: Supplementary Information [file srep23192-s1.doc]
